# Effects of 12-Week Supplementation of a Polyherbal Formulation in Old Adults with Prehypertension/Hypertension: A Randomized, Double-Blind, Placebo-Controlled Trial

**DOI:** 10.1155/2019/7056872

**Published:** 2019-07-14

**Authors:** Tian Shen, Guoqiang Xing, Jingfen Zhu, Yong Cai, Shuxian Zhang, Gang Xu, Yi Feng, Donghua Li, Jianyu Rao, Rong Shi

**Affiliations:** ^1^School of Public Health, Shanghai Jiao Tong University, Shanghai 200025, China; ^2^The Affiliated Hospital and the Second Clinical Medical College of North Sichuan Medical College, Nanchong Central Hospital, Nanchong 637000, China; ^3^Lotus Biotech.com LLC, John Hopkins University-MCC, 9601 Medical Center Drive, Rockville, MD 20850, USA; ^4^Tang Qiao Community Health Service Center, Pudong New District, Shanghai 200127, China; ^5^Department of Pathology and Laboratory Medicine, David Geffen School of Medicine, University of California, Los Angeles, CA 90095, USA; ^6^School of Public Health, Shanghai University of Traditional Chinese Medicine, Shanghai 201203, China

## Abstract

**Background:**

Uncontrolled blood pressure is the leading cause of mortality and disability due to associated cerebral and cardiovascular diseases and kidney failure. More than one-third of the old adult population have hypertension or prehypertension and many of their blood pressure are poorly controlled.

**Objective:**

We hypothesized that plant extracts-based antioxidants may benefit those with prehypertension/hypertension.

**Method:**

One hundred age- and gender-matched healthy older adults were randomly assigned to receive HyperBalance capsules (n=50) or placebo (n=50) at Tang-Qiao Community Health Service Center, Shanghai. Blood pressure and severity scores of hypertension treatment-related symptoms (dizziness, headache, ringing/buzzing in ears, rapid heart rate, and chest tightness) were evaluated before and after the 12-week intervention.

**Results:**

Ninety-eight people completed the study, with 2 dropouts in the placebo group before the end of the study. Forty-one subjects (82%) of the HyperBalance group and 40 subjects (83.3%) of the placebo group had prehypertension (systolic blood pressures (SBP) between 130-139 and diastolic blood pressure (DBP) between 85-89mmHg), and 9 subjects (18%) in the HyperBalance group and 8 subjects (16.7%) in the placebo group had hypertension (≥140/90mmHg) before the intervention. HyperBalance significantly (P<0.01) reduced SBP from 136.18±4.38 to 124.14±3.96 mmHg and reduced DBP from 82.45±2.91 to 80.24±2.41mmHg, respectively, and reversed all 9 hypertension people to normotension or prehypertension state, whereas the placebo moderately reduced SBP from 135.79±4.22 to 132.35±4.656mmHg and reduced DBP from 82.90±3.07 to 82.27±3.01mmHg. All symptom severity scores became significantly lower in the HyperBalance group than in the placebo group after HyperBalance intervention: dizziness (0.82±0.44; vs 2.02±0.64, P<0.01); headache (0.46±0.50; vs 1.81±0.61, P<0.01); ringing/buzzing in ears (0.44±0.50; vs 1.04±0.29, P<0.01); and rapid heart rate and chest tightness (0.30±0.46; vs 0.92±0.28, P<0.01).

**Conclusion:**

Polyherbal supplementation such as HyperBalance could benefit old adults with prehypertension/hypertension and improve treatment-related symptoms. Further studies are needed to validate the current findings.

## 1. Introduction

Uncontrolled high blood pressure is a leading cause of mortality and disability due to cardiovascular disease (CVD) and kidney failure [1]. For example, one in three adults in the US and China have high blood pressure, many of whom remain untreated [2–4]. A recent study in China shows the prevalence of CVD to be 36.0% for prehypertension (high-normal blood pressure) and 30.8% for hypertension, respectively, both of which are correlated with age, gender, alcohol drinking, body mass index (BMI), abdominal obesity, and dyslipidemia [2, 5].

Individuals with prehypertension are characterized with either elevated systolic blood pressure (SBP) ranging from 120 to 139 mmHg or a diastolic blood pressure (DBP) from 80 to 89 mmHg and are at a higher risk of developing hypertension (high blood pressure, with the SBP/DBP readings ≥140/90 mmHg) compared to people with normal blood pressure. Individuals with prehypertension and hypertension, although often asymptomatic (without symptoms), can develop treatment-related symptoms such as dizziness, headaches, visual changes, fatigue, or other nonspecific symptoms after current medical treatment.

Individuals with prehypertension can significantly increase the risk for cardiovascular disorders and kidney problems [6]. It has been reported that a person with prehypertension is 3 times more likely to have a heart attack and 1.7 times more likely to have heart disease than a person with normal blood pressure [7]. Others show that prehypertension is associated with a risk-factor-adjusted hazard ratio for cardiovascular diseases of 2.5 in women and 1.6 in men [8].

Aging, obesity, high salt intake, lack of exercise, stress, depression, and insulin resistance all can contribute to prehypertension/hypertension [2, 9, 10]. More than half of individuals with high blood pressure have a salt-sensitive type of hypertension and progressive renal damage [11, 12]. Low birth weight, maternal smoking, and lack of breast feeding may be risk factors for adult essential hypertension [13].

Recent studies indicate that increased free radical production and diminished activities of antioxidant and nitric oxide (NO) are involved in the development of hypertension [14, 15]. Low-density lipoprotein- (LDL-) cholesterol-induced H_2_O_2_ production is a major causative factor in endothelial perturbation and in the pathogenesis of atherosclerosis. Increased NADPH oxidase activity is also a major source of reactive oxygen species (ROS) in endothelial cells [16, 17]. Similarly, the stroke-prone spontaneously hypertensive rats (SHRSP) exhibit increased salt sensitivity and vascular release of superoxide but decreased total plasma antioxidant capacity [18]. The superoxide release in the SHRSP rats diminishes NO levels that can be reversed or restored by superoxide dismutase (SOD) administration. Thus, increased superoxide generation, decreased antioxidant capacity, elevated vascular inflammatory damage, and renal damage are involved in the progress of prehypertension to hypertension in human and experimental models of salt-sensitive blood pressure [18].

Prevention is considered the best strategy to lower the risk of prehypertension progressing to hypertension. Modification of lifestyle, including low-sodium, high-potassium diet, increasing physical activity, quitting smoking, reducing alcohol consumption, and maintaining a healthy weight, has been recommended [19, 20].

Alternatively, botanical herbs that have historically been used as an essential diet supplement could be potential agents for the management of prehypertension and hypertension [21–23].

For example,* Gastrodia elata* (EG) is one of the most common antihypertension ingredients prescribed by TCM doctors for hypertension [24]. EG contains gastrodin, gastrodigenin, gastrol, 4-Hydroxybenzaldehyde, and 2,4-Bis(4-hydroxybenzyl) phenol as the main active components (Table 1). A recent randomized controlled trial showed that daily intravenous injection of 1,000mg gastrodin for 4 weeks significantly decreased systolic and diastolic blood pressures and reduced plasma endothelin (ET) levels but increased nitric oxide (NO) level in older adults with refractory hypertension who had higher plasma ET and lower level of NO than people with essential hypertension before the treatment [25]. Gastrodin also reduced blood pressure and inhibited angiotensin II (Ang II) and aldosterone (ALD) and the expression of angiotensin type 1 receptor (AT1R) but unregulated peroxisome proliferate-activated receptor gamma (PPAR*γ*) in myocardium of spontaneously hypertensive rats (SHRs) [26]. EG also ameliorated high fructose-induced dyslipidemia, hypertension, and endothelial dysfunction by downregulation of ET and adhesion molecules in the aorta [27]. Moreover, EG significantly restored the impaired vasorelaxation to acetylcholine and the reduced levels of endothelial nitric oxide synthase (eNOS) expression [28, 29]. EG has anti-inflammatory and antiangiogenic activities, through inhibiting LPS-induced expression of inducible nitric oxide synthase (iNOS) and cyclooxygenase-2 [30–32].

Coadministration of multiple medicinal herbal ingredients is a common practice in traditional Chinese medicine. Such practice is to potentiate the bioavailability, activity, and efficacy of the key therapeutic ingredients, and to minimize or antagonize potential toxicities associated with the ingredients.

The primary goal of this exploratory, randomized, placebo-controlled observational study was to determine if administration of polyherbal supplement is beneficial to older adults who have high blood pressure and treatment-related symptoms.

## 2. Methods

The experimental design and procedures of this study were similar to what we recently reported [24, 33, 34].

### 2.1. Samples Size Calculation and Participants Recruitment

This was a randomized, double-blind, placebo-controlled study. Sample size calculation was obtained by the calculation of the estimated experimental study parameters. SBP was chosen as the main outcome, assuming a baseline SBP at about about 135 mm Hg, and the standard deviation at about 7 mm Hg. It was expected that SBP would drop to 130 mm Hg after the intervention:

(1) $$\begin{array}{c} N=\frac{2{\left(Z_{\alpha }+Z_{\beta }\right)}^{2}\sigma^{2}}{d^{2}} \end{array}$$

*σ* is the estimated SD; d is the difference between two groups of continuous variables mean.

According to the two-sided test, *α* = 0.05 *β* = 0.1, *Z*_*α*_** = **1.96 *Z*_*β*_** = **1.282, the calculated sample size N is 41. Assuming a potential dropout rate of 20%, at least 49 people would be required for each group of the study. We enrolled 50 people each for the test group and control group.

The recruitment of the participants into the study was started on December 7, 2011, and completed on December 30, 2011. The last follow-up observation was completed on March 30, 2012, at Tang Qiao Community Health Service Center in Pudong New District in Shanghai, China. Participants were recruited by self-referral in response to media coverage and word of mouth. Witten informed consent was obtained from all participants prior to enrollment into the study. This study was conducted in adherence to CONSORT guidelines (http://www.consort-statement.org/). All study procedures were conducted in accordance with the Helsinki Declaration of 1975 and were approved by the Shanghai Jiao Tong University Medical Center Institutional Review Board. Subjects who met the first and one of the other two following criteria were eligible for the study:

*Inclusion Criteria*. (1) Inclusion criteria were as follows: healthy males or females at least 50 years of age; (2) having current prehypertension (systolic blood pressure 130-139 mmHg or diastolic blood pressure 85-89mmHg); (3) having a history of hypertension (systolic blood pressure 140-159mmHg or diastolic blood pressure 90-99mmHg).

*Exclusion Criteria*. (1) Exclusion criteria were as follows: having diagnosed with any severe medical conditions or complications of the brain, liver, kidneys, heart or lungs, any other organs, or malignant tumors that need medical treatments. The exclusion criteria were to ensure that the study would not be affected by the treatment of other illnesses.

### 2.2. Randomization and Blindness

Participants were randomly assigned to the HyperBalance treatment or placebo treatment. The randomization was performed using a predetermined randomization code, which was generated by a random number generator.

Trial participants and community doctors were both blinded from the treatment (double-blind trial). Of the 100 enrolled participants, 98 participants completed the 12-week follow-up, including 50 subjects in the HyperBalance group and 48 subjects in the placebo group. Two subjects of the placebo group withdrew from the study (due to the objection of the family members).

The participants received similar-looking capsules in color-coded bottles (white bottles for HyperBalance and yellow bottles for placebo). Neither the subjects nor the medical doctors, including the study principal investigator, knew the specific color code until the end of the study. Both the HyperBalance capsules and the placebo (which was mainly composed of flour) were manufactured by Robinson Pharma, Inc. (Costa Mesa, California, USA). Each participant was instructed to take 1 capsule with a meal, two times per day for 12 weeks, and a new batch of supplements was dispensed every month during follow-up sessions.

Table 1 lists the herbal extracts that have known to have supportive properties for blood pressure and are active ingredients of HyperBalance, the formulation used in this study. The key ingredients include extracts of Apocynum* (Apocynum venetum)* leaves, Gou teng* (Uncaria rhynchophylla)*, Eucommia* (Eucommia ulmoides)*, Heal all* (Prunella vulgaris)*, Senna* (Cassia obtusifolia)*, Gastrodia* (Gastrodia elata)*, Wild chrysanthemum* (Chrysanthemum indicum *L*.),* and Rauwolfia* (Rauvolfia yunnanensis)* (Table 1).

### 2.3. Evaluation of Blood Pressure, Treatment-Related Symptoms, and Quality of Life

Changes in blood pressure, treatment-related symptoms including headache, dizziness, ringing/buzzing in ears, rapid heart rate, and chest tightness, and other signs were recorded using a medical questionnaire that included the demographics and medication history of the participants and questionnaire and quality of life evaluation form (SF-36) before and after the completion of the intervention. Measurement of baseline blood pressure and the final blood pressure after the completion of the 12-week follow-up observation were repeated twice separated by 1-week interval. Blood pressure measurement of siting position was conducted by well-trained medical doctors of the community health center, using a mercury sphygmomanometer (Model XJ11D, Shanghai Medical Equipment Corporation, Shanghai). Hypertension treatment-related symptoms, i.e., dizziness, headache, ringing/buzzing in ears, rapid heart rate, and chest tightness, were scored using a self-administered 5-point scale (0=no symptoms; 1=occasional slight symptoms; 2=mild symptoms; 3=moderate symptom; 4=severe symptom). The quality of life was evaluated using the SF-36 that contains 8 domains including physical functioning (PF), role physical (RP), bodily pain (BP), general health (GH), vitality (VT), social functioning (SF), role emotional (RE), and mental health (MH). Items were summed per domain and transformed into scores of 0-100, with higher values representing better functioning. Physical composite score (PCS) and mental composite score (MCS) were computed according to each domain score and the data of Chinese adults model [35]. PCS and MCS were associated with chronic disease, walking and visual abilities, sleep quality, marital status, alcohol consumption,　smoking, filial piety, ethnicity, and dietary habits [36]. All participants were followed up each month in order to check compliance and adverse effects.

### 2.4. Statistics Analysis

EpiData 3.02 software was used for the establishment of the database. SPSS 20 software was used for statistical analysis. Group data were presented as the mean ± s.d. Differences between the HyperBalance and placebo groups were compared using Student's t-test for quantitative variables with normal distribution, or Chi-square test for categorized variables. Ridit scoring test, which is a nonparametric test for comparing two or more sets of ordered qualitative data, was used for evaluating the changes in the symptom severity scores after the intervention. The alpha level of P>0.05 was chosen as being statistically significant. All p-values reported were 2-sided.

## 3. Results

### 3.1. Participants' Demographic Characteristics

The baseline characteristics of age, gender, and histories of alcohol intake, disease, and medication of the participants are shown in Table 2. There were 16 males (32%) and 34 females (68%) in the HyperBalance group, and 22 males (45.8%) and 26 females (54.2%) in the placebo group. The gender distribution between the two groups was not significantly different between the two groups (*χ*^2^=1.97, P>0.05), with females accounting for more than two-thirds of all participants. The average age of all participants was 65.1±7.1, and again no significant difference was found between the HyperBalance group (64.97±7.2 years) and the placebo group (65.28±7.1 years) (t=0.212, P>0.05).

The HyperBalance and placebo groups also showed similar baseline distribution patterns of alcohol drinking (12% vs. 10.4%) (*χ*^2^=0.06, P=0.804), smoking (16% vs. 10.4%) (*χ*^2^=0.664, P=0.415), proportions of prehypertension (82% vs. 83.3%) (*χ*^2^=0.03, p=0.862), hypertension (18% vs 16.7%) (*χ*^2^=0.03, p=0.862), and antihypertension medication (78% vs. 87.5%) (*χ*^2^=1.542, P=0.214). There were three cases of hyperlipidemia in each group (Table 2).

### 3.2. Blood Pressure

The mean values of systolic blood pressure (SBP) and diastolic blood pressure (DBP) of both the HyperBalance group and the placebo group before and after the 12-week intervention are shown in Table 3. There were no baseline differences between HyperBalance and placebo controls before the 12-week intervention in SBP (136.18±4.38 mmHg; vs 135.79±4.22 mmHg, P>0.05), or DBP (82.45±2.91 mmHg vs. 82.90±3.07 mmHg, P>0.05) (Table 3). However, the SBP and DBP became significantly lower in HyperBalance group than in the placebo group after 3 months of HyperBalance intervention (124.14±3.96 mmHg vs. 132.35±4.65 mmHg, P<0.01 and; 80.24±2.41 mmHg vs. 8 82.27±3.01 mmHg, P<0.01, respectively) (Table 3).

### 3.3. Hypertension Treatment-Related Symptoms

There were no baseline differences between HyperBalance group and placebo control in symptom severity scores of dizziness (2.32±0.55; vs 2.42±0.50, P>0.05), headache (2.22±0.62; vs 2.31±0.59, P>0.05), ringing/buzzing in ears (1.02±0.32; vs 1.08±0.28, P>0.05), and rapid heart rate and chest tightness (0.96±0.20; vs 0.98±0.14, P>0.05) (Table 4). However, after 3 months of HyperBalance intervention, all symptom severity scores became significantly lower in the HyperBalance group than in the placebo group: dizziness (0.82±0.44; vs 2.02±0.64, P<0.01); headache (0.46±0.50; vs 1.81±0.61, P<0.01); ringing/buzzing in ears (0.44±0.50; vs 1.04±0.29, P<0.01); and rapid heart rate and chest tightness (0.30±0.46; vs 0.92±0.28, P<0.01) (Table 4), primarily due to a greater symptom severity reduction in the HyperBalance group.

To better evaluate the overall effects of HyperBalance on hypertension-related symptoms, the combined symptom severity scores of dizziness, headache, ringing/buzzing in ears, and rapid heart rate and chest tightness were analyzed. The baseline values in combined symptoms scores were not significant between the HyperBalance and placebo groups (6.56±1.29 vs. 6.91±1.23, P>0.05). However, the combined scores became significant between the two groups after the intervention (2.04±1.47 vs. 5.79±1.41) (P<0.001) (Table 4).

The combined scores of the 4 symptoms were further pooled into 3 subgroups based on levels of symptom improvement (0-2, slight-mild; 3-4, moderate; 5-7 great) for Chi-square analysis. In the HyperBalance group, 48% people showed great symptom severity improvement (5-7 points) and 42% people showed moderate symptom improvement (3-4 points) compared to 2.1% and 14.6% in the placebo group, respectively (P<0.01), whereas 48.0% of the placebo group showed no symptoms improvement (0 point) and 36% showed slight symptom improvement (1-2 points). Ridit scoring test showed a significantly greater improvement in the combined symptom severity scores after the HyperBalance intervention (mean=0.719±0.1.73) than after placebo intervention (0.272±0.183) (t=12.406, P<0.001) (Tables 5 and 6).

### 3.4. Quality of Life

No significant differences were found between the HyperBalance and placebo groups in the baseline values of the different health domains of SF-36 before and after the 3-month intervention. The scores of PF, BP, GH, and VT, however, became significantly increased in the HyperBalance group after the 3-month intervention. The scores of POS and MCS also became significantly higher in HyperBalance and placebo groups after the 3-month intervention, suggesting improved quality of life in the HyperBalance group (Table 7).

## 4. Discussion

Prehypertension and hypertension each affect about 30% of the world adult population and increase the risk of incidents of CVD [3, 5, 37]. According to a 7-year follow-up study of 25,079 prehypertensive people, the annual progression rate of prehypertension to hypertension is more than 5.5% [38]. Prehypertension is also a major risk for heart attacks, strokes, congestive heart failure, kidney failure, and cognitive decline [39–41]. Most prehypertensive people do not receive effective interventions [42, 43]. One reason is that many people do not like to take medication to control prehypertension and/or hypertension due to the perceived side effects and potential addiction associated with the drugs [44].

In this exploratory observational study, the effectiveness of 12-week dietary supplementation of a polyherbal formulation, HyperBalance, was evaluated in older adults with prehypertension or with a history of hypertension and drug treatment. The results show that HyperBalance was significantly more effective than placebo in improving blood pressure, resulting in an average reduction of 12.04 mm gH for SBP and a reduction of 2.21 mm gH for DBP compared to the 3.43 mmHg reduction for SBP and 0.63 mmHg reduction for DBP by placebo. Moreover, HyperBalance was also more effective than placebo in reducing drug treatment-related symptoms in these old adults.

HyperBalance appears to have improved blood pressures to some degree in all participants, including the 23 of the 41 prehypertensive people who were reversed to normal blood pressure and the 9 hypertensive people who were reversed to prehypertension status. In comparison, only 1 of the 40 prehypertensive people of the placebo group was reversed to normal blood pressure and only 4 of the 8 hypertension people showed reduced blood pressure. Furthermore, a greater proportion of the HyperBalance group showed greater improvement in drug treatment-related symptoms including dizziness, headache, ringing/buzzing in ears, and rapid heart rate and chest tightness, as well as quality of life.

These results suggest that that supplementation of polyherbal ingredients such as HyperBalance could benefit people with prehypertension/hypertension and treatment-related symptoms. Although the underlying mechanism of HyperBalance remains unknown, each of the active polyherbal ingredients could have synergistically contributed to the efficacy of HyperBalance.

Our study also shows that the herbal ingredients of HyperBalance share similar yet distinct antioxidant, anti-inflammatory, antiproliferation, antisuperoxide, and NO-promoting properties. Their potential actions are in agreement with the current knowledge that accelerated inactivation of NO and SOD, and elevated renal damage due to increased norepinephrine, angiotensin II, endothelin, and superoxide anion, increased vascular smooth muscle cell proliferation, decreased antioxidant capacity, and are related to the hypertrophy and reduction in outer diameter of the arteries and the development of hypertension [18]. Meanwhile, antioxidant treatment reduces arterial pressure, aortic superoxide production, and renal inflammation in DOCA-salt rats and decreases blood pressure and aortic superoxide release and increases bioactive nitric oxide in SHR stroke-prone rats [15].

*Chrysanthemum indicum* Linne (CIL) contains flavonoids, 1,8-cineole, camphor, borneol, and bornyl acetate as the main active compounds [45, 46]. Intravenous administration of CIL extract at the dosage of 5-20 mg/kg decreased aortic blood pressure but increased coronary blood flow, renal blood flow, left ventricular dP/dt, and heart rate in the dog in a dose-dependent manner [47]. CIL directly and uniformly induced coronary and systemic vasodilation with renal vasoconstriction [48]. CIL extract inhibited the inflammatory response by suppressing NF-kappa B and MAPKs activation in lipopolysaccharide-induced RAW 264.7 macrophages [49]. Pretreatment of CIL attenuated neuronal damage/death in the brain after cerebral ischemia/reperfusion via an anti-inflammatory approach [50].

The main active ingredients of* Apocynum venetum* leaves (AVLE) include apocynin, flavonol glycosides, quercetin, triacontanol, beta-sitosterol, hyperin, and long-chain fatty acids (Table 1). AVLE has blood pressure effects in spontaneously hypertensive rats (SHR), renal hypertensive rats, and NaCl-induced hypertensive rats, due to amelioration of the kidney dysfunctions, inhibition of ROS, and oxidation of low-density lipoprotein (LDL) [18, 51, 52]. AVLE exerts its effect on blood pressure via dilating the blood vessels in an endothelium- and concentration-dependent manner with optimal effect seen at 10 *μ*g/mL, primarily due to its NO-releasing, superoxide-scavenging and NADPH oxidase inhibiting properties [53]. AVLE also possesses antioxidant and anti-lipid peroxidation effects. Apocynin increases glutathione synthesis and activates AP-1 in alveolar epithelial cells and prevents ischemia-reperfusion lung injury [54, 55]. Deoxycorticosterone acetate- (DOCA-) salt hypertension and venoconstriction are characterized by low renin/angiotensin but increased arterial superoxide levels, arterial endothelin-1 (ET-1), NADPH oxidase activation, and superoxide generation, all of which can be reversed by AVLE [56]. AVLE and its purified components can strongly inhibit the formation of advanced glycation end products (AGEs), which are involved in the pathogenesis of diabetic vascular complications and atherosclerosis [57].

*Uncaria rhynchophylla* has long been used in Chinese medicine to treat cardiovascular disorder, hypertension, headaches, and other cognitive concerns [58, 59]. Various compounds including indole alkaloids (rhynchophylline and isorhynchophylline, triterpenes), flavonoids, phenols, and phenylpropanoids have been isolated with indole alkaloids as the main efficacy component for hypertension. Rhynchophylline and isorhynchophylline exert endothelium-independent vasodilatory effects that are mediated by L-type Ca2+ channels [60]. Isorhynchophylline can inhibit endotoxemic, vascular smooth muscle cell proliferation (a process that involves the development of blood pressure) through increased NO production and cell cycle regulation [61, 62].

*Eucommia ulmoides* (EU) contains various chemical constituents such as lignans, iridoids, phenolics, steroids, and flavonoids that have multiple antioxidant, anti-inflammatory, antiallergic, antimicrobial, anticancer, antiaging, cardioprotective, and neuroprotective properties [63].* Eucommia ulmoides* lignans are effective in lowering blood pressure, protecting against hypertensive renal injury in spontaneous hypertensive rats, and inhibiting aldose reductase (AR) overexpression in the kidney [64].* Eucommia* lignans inhibit angiotensin II- (Ang II-) induced proliferation of rat mesangial cells via regulating cell cycle-related genes P21 and P27 [65] and exert their antihypertensive effects via regulating NO and renin-angiotensin system (RAS) and direct artery relaxing effect [66]. Cellular proliferation is an important pathological factor in hypertensive renal injuries, and increased Ang II expression was essential for target-organ damage during hypertension. Ang II is the main effective peptide in the RAS and is a key mediator in the development of hypertensive nephropathy [67].

*Prunella vulgaris* (*P. vulgaris*) leaves (PVL) have been widely consumed as a tea ingredient since ancient times for its antiviral, antibacterial, anti-inflammatory, antiallergic, antioxidative, antitumor, antihypertensive, antihyperglycemic, and immunoregulatory properties [68–71]. Triterpenoic acids, flavonoids, phenolics, and diterpene are the major active compounds. APL treatment (100 and 200 mg/kg/day) for eight weeks markedly lowered systolic blood pressure, blood glucose, total plasma cholesterol, triglyceride, LDL-cholesterol, malondialdehyde, and TGF-beta1 but improved total NO level, HDL-cholesterol level, and diabetic vascular dysfunction in high-fat high cholesterol mice with type 2 diabetes [72].* P. vulgaris* dose-dependently ameliorated vascular constriction of aortic rings induced by acetylcholine or SNP [72]. Ursolic acid in PVL stimulates blood circulation via inhibition of Na+/K+ -ATPase [73]. PVL also dose-dependently inhibited high glucose- (HG-) induced expression of cell adhesion molecules (ICAM-1, VCAM-1, and E-selectin) in human umbilical vein endothelial cells, inhibited HG-induced adhesion, and suppressed p65 NF-kappa B activation in HL-60 monocytic cells, as well as inhibiting the formation of intracellular reactive oxygen species (ROS) [74] and the proliferation of vascular smooth muscle cells [75]. PVL inhibits proinflammatory cytokines, NF-kappa B, and mast cell-derived immediate-type allergic reactions [76] and shows antihyperglycemic activity in streptozotocin-induced diabetic mice [77]. PVL extract stimulates macrophage phagocytic activity, nitric oxide (NO) production, and cytostatic activity and induces gene expression and macrophage-related TNF-alpha, IL-1beta, and IL-6 [78]. Rosmarinic acid (RA) of PVL inhibits inflammatory response, LPS-induced cyclooxygenase-2 (COX-2), and iNOS protein expression [79].

Senna (*Cassia obtusifolia*) oral administration of obtusifolin extracts significantly ameliorated high-fat diet-induced changes in body weight, total cholesterol, triglyceride, low-density lipoprotein cholesterol, high-density lipoprotein cholesterol, and malondialdehyde but increased serum superoxide dismutase and nitric oxide [80]. Naphthopyrone glycosides from* Cassia obtusifolia* have antiallergic effect by inhibiting histamine release [33], and anthraquinones from seeds of* Cassia obtusifolia* are potent inhibitors of platelet aggregation [34].

*Rauwolfia yunnanensis *contains alkaloids (reserpine, rescinnamine, reserpiline), ajmaline, aricine, corynanthine, deserpidine, lankanescine, rauwolscine, etc. as the main active compounds [81]. Rserpine is as effective as other first-line antihypertensive drugs in reducing SBP [82, 83] and it was widely used in the 1950s for treatment of hypertension. Reserpine can effectively reduce blood pressure, depress the activity of the central nervous system, and act as hypnotics [25]. Now only low dose of* Rauwolfia* is used as a cotreatment of hypertension due to its side effects such as oxidative damage to liver and other organs and depression [84].

So far, few studies compared single medical herbal ingredients with polyherbal formulation on prehypertension although combination therapy of different antihypertensive agents often results in better efficacy than single-pills therapy [85]. One recent study showed that 6-month supplementation of a mostly* Rauwolfia* serpentine formulation (plus several other herbal-mineral ingredients) in 30 adults with prehypertension or stage I hypertension significantly increased serum potassium (p < 0.05) but significantly decreased systolic blood pressure (p < 0.0001) and diastolic blood pressure (p < 0.0001) [25]. While no serious adverse events were reported, 30% of the participants withdrew from the citing potential side effects including nasal congestion or fatigue as the most common side effects [25].

One early study examined 295 moderate arterial hypertension patients with the diastolic pressure under 110 mm Hg [29]. Of these, 103 patients who received early hypotensive therapy of* rauwolfia* drugs (13 patients also received additional hypothiazide) showed decrease in arterial blood pressure to normal level and an 8-year increase in mean lifespan compared to the 80 patients who had not received any hypotensive drugs. Furthermore, the number of fatal brain strokes and myocardial infarctions decreased almost 2-fold in the treatment group.

In this study, a significant proportion (82.7%) or 81 of the 98 participants received the antihypertension medicine amlodipine tablet for a period between 3 and 36 months.* Amlodipine* is a calcium channel blocker that dilates (widens) blood vessels and improves blood flow but it may also induce treatment-related symptoms. It is known that antihypertension medicines can induce different side effects such as dizziness, weakness, fatigue, shortness of breath, difficulty breathing, and chest pain induced by beta-blockers, headache, dizziness, fainting, reduced kidney function induced by ACE Inhibitors, and affected muscular function of the heart by diuretics [72, 74, 75, 80, 86–92]. Further studies could validate the beneficial effects of polyherbal ingredients in managing prehypertension/hypertension and treatment-related symptoms.

There are limitations of this study. The sample size of this study is too small to reach a conclusion. Because risk factors for prehypertension/hypertension are heterogeneous and potentially affected by a range of genetic and environmental factors including lifestyle, daily activity/exercise, genotype, age, obesity, diabetes, stress, education level, inflammation, alcohol intake, and smoking, it is difficult to know the optimal dose of individual ingredients for a most effective formulation. Further studies could control these factors to allow a better understanding of the mechanism of polyherbal intervention.

## 5. Conclusion

In conclusion, 12-week supplementation of polyherbal ingredients such as HyperBalance could benefit older adults with prehypertension/hypertension in improving blood pressures and treatment-related symptoms. Further studies of long-term supplementation of polyherbal medicine are needed to validate the current findings.

## Figures and Tables

**Table 1:** Information of the key ingredients of HyperBalance.

Ingredients	Main active compounds	Known effects & possible mechanisms of actions
Apocynum (Apocynum venetum)	flavonol glycosides	protect LSP-induced heart, lung, kidney and liver injuries
	quercetin, hyperin	selectively inhibits neutrophil superoxide and peroxynitrite production, yet with intact bactericidal activity, preventive of LPS-induced emphysema
	plant-phenol (apocynin)	affects blood pressure, anti-inflammatory, anti-ischemic, anti-arthritic, antihyperlipidemic, anti-diabetic, anti-aging, antithrombic, anti-stress, anti-advanced glycation end products
	triacontanol, beta-sitosterol, lupeol	Exert long-lasting endothelium-dependent relaxant effect via stimulating nitric oxide production, ROS-scavenging Src/PI3K/Akt Pathway
	scopoletin and isofraxidin.	
	Isofraxidin and hyperin	
Gou Teng (Uncaria rhynchophylla)	indole alkaloids	
	rhynchophylline, isorhynchophylline	Act against hypertension, asthma, cancer, cirrhosis, diabetes, stroke, rheumatism, bradycardia, arrhythmia, sedation, neurodegeneration, epileptic seizures, drug addiction, and cerebral ischemia, coagulation, endotoxemic
	triterpenes	protect cardiovascular/neural/neuroglial cells, modulate calcium and potassium ion channels and neurotransmitter transporters
	flavonoids	inhibit angiotensin II induced vascular smooth muscle cells proliferation, increased NO content and NOS activity
	phenols	Rhy and Iso rhy have endothelium-independent vasodilatory effects via L-type Ca2+ channels and other Ca2+ pathways
	phenylpropanoids	
Eucommia (Eucommia ulmoides)	lignans	have antihypertensive effect via regulating NO and renin-angiotensin system and direct artery relaxing effect.
	flavonoids, iridoids	have antioxidant, anti-inflammatory, antiallergic, antimicrobial, anticancer, antiaging, cardioprotective, and neuroprotective properties
	polysaccharides	protect against blood pressure renal injury, inhibit renal aldose reductase overexpression, inhibit Ang II-induced mesangial cells proliferation via cycle-related genes P21 and P27, and Bax
	terpenes	
Heal all (Prunella vulgaris)	triterpenoic acids,	anti-hypertensive, anti- allergic, anti-hyperglycemic, anti-hyperlipidemic, anti-hyper cholesterol,
	flavonoids	anti-inflammation via inhibition of COX-2 and iNOS expression, ROS-scavenger, anti-proliferation of vascular smooth muscle cells
	phenolics	stimulate blood circulation via inhibition of Na+/K+ -ATPase, inhibit inflammatory cytokines,
	diterpene, polysaccharides	inhibit ROS production, restore NO production
Senna (Cassia obtusifolia)	pyridoxal, pyridoxamine, pyridoxine	anti-hyperlipidemic, antihypercholesterol, anti-allergic via inhibiting histamine release,
		improve SOD and NO activity, inhibit platelet aggregation
Gastrodia (Gastrodia elata)	gastrodin	affects blood pressure via reducing plasma endothelin levels, inhibiting angiotensin II, aldosterone and angiotensin type 1 receptor but increasing NO and PPAR*γ* levels
	gastrodigenin	restores the impaired vasorelaxation and reduced eNOS
	4-Hydroxybenzaldehyde	exerts anti-inflammatory and anti-angiogenic activities through inhibiting LPS-induced expression of iNOS and COX-2
	2,4-Bis(4-hydroxybenzyl) phenol	
Wild Chrysanthemum (Chrysanthemum indicum)		dose-dependently decreased aortic blood pressure but increased coronary blood flow, renal blood flow,
		induces coronary and systemic vasodilation with renal vasoconstriction
		habits the inflammatory response and ischemic injury by suppressing NF-kappa and MAPKs activation
Rauwolfia (Rauvolfia yunnanensis)	indole alkaloids	can significantly reduce systolic blood pressure to the same degree as other first-line blood pressure maintenance drugs
	(reserpine, rescinnamine, reserpiline)	
	ajmaline, aricine, corynanthine	
	deserpidine lankanescine rauwolscine	

**Table 2:** Demographics and medical history of the participants.

Items	Male			Female			Total		
	HyperBalance	Placebo	Combined	HyperBalance	Placebo	Combined	HyperBalance	Placebo	Combined
Gender, N (% of subtotal)	14 (29.8)	17 (33.3)	31 (31.6)	33 (70.2)	34 (66.7)	67(68.4)	50 (100)	48 (100)	98 (100)
Age, year (mean ± s.d)	65.35 ± 7.12	67.18 ±7.4	66.41 ± 7.25	64.79 ± 7.29	63.67 ± 6.53	64.31 ± 6.94	64.97 ± 7.17	65.28 ± 7.09	65.12 ± 7.10
Age, year (range)	52.87-82.80	47.28-83.47	52.87-84.46	53.18-84.86	49.03-88.43	47.28-88.43	52.87-84.86	47.28-88.43	47.28-88.43
Smoking (Yes/Total, %)	8/50 (16.0)	5/48 (10.5)	13/98 (13.3)	0(0)	0(0)	0(0)	8/50 (16.0)	5/48 (10.5)	13/98 (13.3)
Alcohol drinking (Y/T, %)	6/50 (12.0)	5/48(10.5)	11/98(11.2)	0(0)	0(0)	0(0)	6/50 (12.0)	5/48 (10.5)	11/98 (11.2)
Hyperlipidemia (Y/T, %)	1/50 (2.0)	0(0)	1/98(1.0)	2/50(4.0)	3/48 (6.25)	5/98(5.1)	3/50 (6.0)	3/48 (6.25)	6/98 (6.12)
Prehypertension (Y/T, %)	12/50 (24)	18/48 (37.5)	30/98 (30.6)	29/50 (58)	22/48 (45.8)	51/98 (52.1)	41/50(82)	40/48(83.3)	81/98 (82.7)
Hypertension (Y/T, %)	3/50 (6)	4/48 (8.3)	7/98 (7.1)	6/50 (12)	4/48 (8.3)	10/98 (10.2)	9/50(18)	8/48(16.7)	17/98 (17.3)
History of anti-hypertension	12/50 (30.8)	20/48(47.6)	32/98(39.5)	27/50(69.2)	22/48(52.4)	49/98(60.5)	39/50 (78)	42/48 (87.5)	81/ 98 (82.7)
Medication (Y/T, %)									

**Table 3:** Blood pressure before and after 12-week of HyperBalance/Placebo intervention (mean ± s.d).

Blood Pressure (mmHg)	Before intervention	After intervention
Systolic blood pressure (SBP)		
HyperBalance	136.18±4.38	124.14±3.96*∗*
Placebo	135.79±4.22	132.35±4.65*∗*
t / P value	0.45/ 0.66	9.43/ <0.001^**++**^
Diastolic blood pressure (DBP)		
HyperBalance	82.45±2.91	80.24±2.41*∗*
Placebo	82.90±3.07	82.27±3.01*∗*
t / *P* value	0.74/ 0.46	3.68/ <0.001^**++**^

*∗*, P<0.05, comparison of baseline with the post-intervention values within each treatment group;

^**++**^, P<0.001, difference between Hyperbalance and placebo groups after the 12-week intervention

**Table 4:** Changes in individual and combined severity scores of anti-hypertension medicine treatment-related symptoms (mean ± s.d) after 12 weeks of HyperBalance/placebo intervention.

Group	Before intervention	After intervention	Difference between before and after intervention	t / P value
Dizziness				
HyperBalance	2.32 ± 0.55	0.82±0.44*∗*	1.50±0.54	19.498/<0.001
Placebo	2.42 ± 0.50	2.02±0.64*∗*	0.40±0.57	4.779/<0.001
t / *P* value	0.91/ 0.37	10.94/ <0.001	9.778/<0.001^**++**^	
Headache				
HyperBalance	2.22 ± 0.62	0.46±0.503*∗*	1.76±0.48	26.124/<0.001
Placebo	2.31 ± 0.59	1.81±0.607*∗*	0.50±0.58	5.937/<0.001
t / *P* value	0.76/ 0.45	12.03/ <0.001^**++**^	11.683/<0.001^**++**^	
Ringing/buzzing in ears				
HyperBalance	1.02 ± 0.32	0.44±0.501*∗*	0.58±0.54	7.624/<0.001
Placebo	1.08 ± 0.28	1.04±0.29	0.04±0.20	1.430/0.159
t / *P* value	1.04/ 0.30	7.24, <0.01	6.608/<0.001^**++**^	
Rapid heart rate and chest tightness				
HyperBalance	0.96 ± 0.20	0.30±0.46*∗*	0.66±0.48	9.753/<0.001
Placebo	0.98 ± 0.14	0.92±0.28	0.06±0.24	1.770/0.083
t / *P* value	0.55, 0.59	7.94, <0.001	7.828/<0.001^**++**^	
Combined symptom scores				
HyperBalance	6.52±1.27	2.02±1.46*∗*	4.50±1.36	23.414/ <0.001
Placebo	6.79±1.07	5.79±1.41*∗*	1.00±1.29	5.378/ <0.001
t / *P* value	1.145/0.255	12.967/<0.001	13.074/<0.001^**++**^	

*∗*, P<0.05, comparison of baseline with the post-intervention values within each treatment group;

^**++**^, P<0.001, comparison of the difference between Hyperbalance and placebo groups after the 12-week intervention.

**Table 5:** Different impact of HyperBalance and placebo on combined blood pressure severity scores.

Group	Mild change (0-2) *∗*	Moderate (3-4) *∗∗*	Large change (5-8) *∗∗∗*
HyperBalance, n (%)	5(10)	21(42)	24(48)
Placebo (%)	40(83.3)	7(14.6)	1(2.1)
*χ* ^2^/P	55.364/<0.01		

*∗*0-2 points: none to mild symptom severity score reduction

*∗∗*3-4 points: moderate symptom severity score reduction

*∗∗∗*5-8 points: large symptom severity score reduction.

**Table 6:** Different impact of HyperBalance and placebo on combined blood pressure severity scores.

Score Change	HyperBalance		Placebo		Total	
	N	%	N	%	N	%
0	0	0	23	48	23	23
1+	0	0	8	17	8	8
2+	5	10	9	19	14	14
3+	5	10	6	13	11	11
4+	16	32	1	2	17	17
5+	9	18	0	0	9	9
6+	13	26	1	2	14	14
7+	2	4	0	0	2	2
Total	50	100	48	100	98	100

t (Ridit scoring test)	12.406					
P value	<0.001					

**Table 7:** Changes in SF-36 scores after the 3 month-intervention.

Items	Before intervention		After intervention	
	HyperBalance	Placebo	HyperBalance	Placebo
PF	90.7±3.2	91.15±2.36	91.1±2.73*∗*	91.25±2.19
RP	100±0	100±0	100±0	100±0
BP	78.02±8.16	79.21±6.79	83.22±11.38*∗∗*	80.17±8.83
GH	49.78±4.97	50.6±5.39	51.88±8.8*∗∗*	50.9±8.2
VT	86.7±3.29	87.19±3.98	87.4±3.07*∗*	87.4±3.42
SF	99.25±3.92	100±0	99.75±1.77	100±0
RE	100±0	100±0	100±0	100±0
MH	87.36±4.6	88.33±5.77	87.76±4.15	88.25±5.11
HT	48.5±6	50.52±3.61	48.5±6	50±5.16
PCS	66.88±3.75	76.8±4.88	67.53±3.71*∗∗*	75.63±4.41*∗∗*
MCS	68.24±3.37	69±4	81.8±2.3*∗∗*	82.39±3.42*∗∗*

*∗*P<0.05, within treatment group changes after 3-month intervention

*∗∗* P<0.01, within treatment group changes after 3-month intervention.

## Data Availability

The raw data is not publicly available. However, the raw data could be available from the corresponding authors upon reasonable request and with permission of the study sponsor.

## Abbreviations

ALD: Aldosterone
Ang II: Angiotensin II
AVLE: * Apocynum venetum* leaves
BMI: Body mass index
BP: Bodily pain
CIL: * Chrysanthemum indicum* Linne
COX-2: Cyclooxygenase-2
CVD: For cardiovascular disorders
DBP: Diastolic blood pressure
DOCA: Deoxycorticosterone acetate
EG: * Gastrodia elata*
eNOS: Endothelial nitric oxide synthase
E-selectin: A cell surface molecule involved in immune adhesion and cell trafficking
ET: Endothelin
EU: * Eucommia ulmoides*
GH: General health
HG: High glucose
HyperBalance: A polyherbal extracts-based formulation
ICAM-1: Intercellular adhesion molecule 1 (cell adhesion molecules)
iNOS: Inducible nitric oxide synthase
LDL: Low-density lipoprotein
MAPK: Mitogen-activated protein kinase
MCS: Mental composite score
MH: Mental health
NADPH: Nicotinamide Adenine Dinucleotide phosphate (reduced form)
NF-kappaB: Nuclear factor kappa-light-chain-enhancer of activated B cells
NO: Nitric oxide
PCS: Physical composite score
PF: Physical functioning
PPAR*γ*: Peroxisome proliferate-activated receptor gamma
PVL: * Prunella vulgaris* (*P. vulgaris*) leaves
RA: Rosmarinic acid
RE: Role emotional
ROS: Reactive oxygen species
RP: Role physical
SBP: Systolic blood pressures
SF: Social functioning
SHRSP: Stroke-prone spontaneously hypertensive rats
SOD: Superoxide dismutase
TCM: Traditional Chinese medicine
TGF-beta1: Transforming growth factor beta1
VCAM-1: Vascular cell adhesion molecule 1
ventricular dP/dt: Measures of left ventricle (LV) pressure or global contractility
VT: Vitality.
